# Three-dimensional-bioprinted stiff matrix triggers PDAC radioresistance through histone H3 lysine 18 lactylation (H3K18la) potentiates RAD51 activation

**DOI:** 10.1093/rb/rbag071

**Published:** 2026-04-10

**Authors:** Xue Zhang, Hongyu Zhu, Zihan Shi, Yifei Lu, Mingyue Chang, Yan Li, Yahong Zhao, Yumin Yang, Yibing Guo

**Affiliations:** Research Center of Clinical Medicine, Affiliated Hospital of Nantong University, Nantong University, Nantong, Jiangsu 226001, China; Jiangsu Key Laboratory of Tissue Engineering and Neuroregeneration, Co-Innovation Center of Neuroregeneration, Nantong University, 226001, P. R. China; Research Center of Clinical Medicine, Affiliated Hospital of Nantong University, Nantong University, Nantong, Jiangsu 226001, China; Department of Trauma Center, Affiliated Hospital of Nantong University, Nantong, Jiangsu 226001, China; Research Center of Clinical Medicine, Affiliated Hospital of Nantong University, Nantong University, Nantong, Jiangsu 226001, China; Department of Gastroenterology, Affiliated Hospital of Nantong University, Nantong, Jiangsu 226001, China; Research Center of Clinical Medicine, Affiliated Hospital of Nantong University, Nantong University, Nantong, Jiangsu 226001, China; Jiangsu Key Laboratory of Tissue Engineering and Neuroregeneration, Co-Innovation Center of Neuroregeneration, Nantong University, 226001, P. R. China; Research Center of Clinical Medicine, Affiliated Hospital of Nantong University, Nantong University, Nantong, Jiangsu 226001, China; Jiangsu Key Laboratory of Tissue Engineering and Neuroregeneration, Co-Innovation Center of Neuroregeneration, Nantong University, 226001, P. R. China; Jiangsu Key Laboratory of Tissue Engineering and Neuroregeneration, Co-Innovation Center of Neuroregeneration, Nantong University, 226001, P. R. China; Research Center of Clinical Medicine, Affiliated Hospital of Nantong University, Nantong University, Nantong, Jiangsu 226001, China

**Keywords:** pancreatic ductal adenocarcinoma, radioresistance, 3D bioprinting, matrix stiffness, histone lactylation

## Abstract

Radiotherapy improves the survival and life quality of individuals diagnosed with locally advanced or terminal pancreatic ductal adenocarcinoma (PDAC). Nevertheless, the ubiquitous of radioresistance resulted in ineffective outcome, which commonly observed the recurrence and malignant progression within the radiation target area. Our clinical observation has shown that PDAC cells resident in rigid tumor niche, therefore, to illuminate the underlying mechanism from the biomechanical perspective is of significant importance. To this end, PDAC models with tunable mechanical properties were constructed through 3D-bioprinted gelatin methacryloyl (GelMA) hydrogel, which enabled the precise manipulation of cellular behavior under physiological stiffness scopes. The effect of matrix stiffness on radiation tolerance of PDAC cells subjected to distinct X-ray radiation was assessed, and the results validated that stiff matrix promoted radioresistance. To elucidate the in-depth mechanism underlying this phenomenon, enrichment analysis was performed on DEGs between soft and stiff groups treated with 4 Gy X-ray radiation. The results showcased that glycolysis process was prominent enriched, further experiment demonstrated that matrix stiffness promoted the glycolysis level and lactate accumulation. The stiff group reinforced histone H3 lysine 18 lactylation level, and the inhibition of histone lactylation efficiently suppressed PDAC radioresistance. shRAD51 assay corroborated that H3K18 lactylation-driven RAD51 transcriptional activation, which established a causal link to radioresistance. Overall, our research shed light on the matrix stiffness mediated histone lactylation, which exerted essential role in PDAC radioresistance and provided insight into future radiotherapy from the perspective of biomechanics.

## Graphical abstract

**Graphical Abstract:**
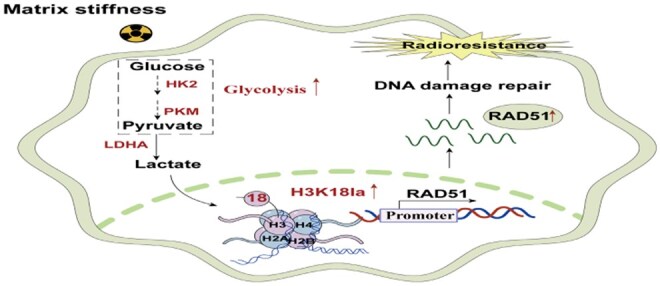

## Introduction

Multimodal therapies including radiotherapy (RT) and chemotherapy have commonly been applied in PDAC, the most aggressive and fatal malignancy tumor. The patients exhibited an inferior 5-year survival rate partly due to therapy resistance. During the development and progression of PDAC, the pronounced rigid extracellular matrix (ECM) was observed. Currently, the function and potential mechanism of matrix stiffness on chemoresistance have attracted extensive attention and explored in depth. For instance, high stiffness upregulated drug efflux pumps through CD44-hyaluronic acid interaction to trigger PDAC chemotherapy resistance [1]. Stiffening hydrogel mediated drug resistance through promoting the epithelial-to-mesenchymal transition and the enrichment of CD133^+^/CXCR4^+^ subpopulation [2]. Our prior investigations demonstrated that matrix stiffness modulated PDAC chemoresistance via autophagy and metabolic reprogramming [3, 4]. While for PDAC patients with locally advanced or terminal period, radiotherapy provided significant survival benefit. However, radioresistance remains the primary impediment to boosting RT efficacy, while the combination with other therapeutic strategies further heightens the outcomes. Zoledronic acid enhanced radiation through the inhibition of farnesyl diphosphate synthase (FDPS), a cholesterol biosynthesis pathway enzyme. The concurrent with RT and cyclin-dependent kinase-1 (CDK1) inhibition reinforced the treatment of PDAC [5]. Diminishing ECM stiffness with the inhibition of lysyl oxidase augmented radiotherapy efficacy of liver cancer [6]. Soft ECM promoted breast cancer therapy resistance by suppressing JNK-mediated apoptosis and enhancing NF-κB survival signaling [7]. In nasopharyngeal carcinoma, soft ECM exhibited enhanced radiation resistance through increased tumor stem cell proportion and G1/S phase arrest-driven proliferation [8]. Accordingly, ECM stiffness is strongly connected with radioresistance, while the underlying mechanisms relate to PDAC still need to be explored.

PDAC cells resident in three-dimensional (3D) niche *in vivo*, which endure forces from all directions. Three-dimensional-bioprinting technology can resemble the pathophysiological characteristics of *in vivo* setting with precise architecture and matrix stiffness, making it suitable for exploring the effect on cellular behavior [9, 10]. The photocurable GelMA hydrogel was broadly leveraged in 3D bioprinting due to the fine biocompatibility, adjustable physicochemical traits and excellent plasticity [11]. For example, GelMA enhanced osteogenesis through the mitochondrial phosphoenolpyruvate carboxykinase (PCK2)-mediated glycolysis in a three-dimensional ambience [12]. Three-dimensional-bioprinted osteosarcoma models with distinct matrix stiffness showcased that softer matrix promoted proliferation, migration and drug resistance [13]. While for bioprinted gastric cancer models with precise ECM stiffness based on the gastric tissue-specific bioink, which exhibited augmented malignant phenotypes, including cellular interactions and drug resistance [14].

Abnormal metabolism, such as glycolysis, is a major hallmark of carcinoma. The byproduct lactate triggered histone lysine lactylation to stimulate gene transcription from chromatin, which was a new epigenetic modification [15]. Histone lactylation regulated numerous biological processes, including oncogenic development, disease progression, immune escape and resistance [16]. For instance, the immunosuppressive properties of monocyte-derived macrophages were boosted by histone lactylation, thereby contributing to tumor immune escape [17]. Histone lactylation reinforced resistance to bevacizumab via elevating RUBCNL expression, an autophagy enhancer protein in colorectal cancer [18]. H3K14la activated the NEDD4 ubiquitin ligase, leading to ubiquitination-mediated degradation of the tumor suppressor PTEN, thus, promoting HCC chemoresistance [19]. H4K12la upregulated the glutathione synthesis rate-limiting enzyme GCLC to inhibit ferroptosis, and enhanced cancer stem cells’ oxaliplatin resistance [20]. The elevated lactate level and histone H3 lysine 18 lactylation (H3K18la) elicited the upregulation of ACAT2 in pancreatic cancer cells, reinforcing the link between metabolic reprogramming and histone modification-driven gene expression [21]. H3K18la activated transcription factors YBX1 and YY1, coordinating with enhanced glycolytic metabolism to drive cisplatin resistance [22]. The radiotherapy increased the glycolysis and lactate secretion of PDAC [23]. Lactic acid accumulation driven H4K12la-induced RAD23A to strengthen niraparib resistance in ovarian cancer [24]. Additionally, H3K9la stimulated RAD51 expression to drive homologous recombination (HR) repair and ultimately promote cisplatin resistance [25]. By orchestrating HR repair, the evolutionarily conserved protein RAD51 is integral to DNA double-strand breaks (DSB) repair and radioresistance, facilitating replication fork restart and error-free restoration of DSBs [26]. RAD51 expression was elevated in glioblastoma tissues following radiotherapy, and correlated with significantly diminished survival [27]. However, whether and how matrix stiffness mediated PDAC radioresistance through histone lactylation warrants further investigation.

Taken together, our research aims to elucidate the exact role and underlying mechanism between ECM stiffness and radioresistance. First, PDAC models with distinct matrix stiffnesses were fabricated through 3D-printed GelMA hydrogel. Furthermore, RNA-sequencing and enrichment analysis were conducted to explore the potential mechanisms. Finally, the relationship among H3K18 lactylation, RAD51-mediated DNA repair and radioresistance was illustrated. Collectively, our research aims to uncover the underlying mechanism of radioresistance in terms of biomechanics-regulated epigenetic modification, providing a potential target for radiotherapy strategies.

## Materials and methods

### Cell cultivation

The human pancreatic cancer MIA-PaCa2 and CFPAC-1 cell lines were obtained from Chinese Academy of Sciences Cell Bank. The Dulbecco’s Modified Eagle’s Medium (DMEM, BioChannel Biological Technology Co., Ltd.) with the addition of 10% fetal bovine serum (FBS, BioChannel Biological Technology Co., Ltd.) were utilized for the cultivation. The 1% sodium pyruvate and 3% horse serum were supplemented to meet the specific nutritional requirement of MIA-PaCa2. Both cell lines were routinely propagated under 5% CO_2_ at 37°C.

### Rheological testing

The measurement parameters for temperature sweep: 5–45°C, 1 Hz frequency and 1% oscillatory strain. To illustrate the shear-thinning property of GelMA, the measurement condition for rheological shear rate sweep test: 1–1000 rad s^−1^, 37°C. The range of 0.1–10 Hz at the fixed 1% strain for frequency sweep, while time sweep (10 min) was carried out at 1 Hz frequency and 1% strain. All tests were measured on a rheometer (Haake RS6000, Thermo Scientific, USA) and repeated at least thrice.

### Three-dimensional bioprinting

Both GelMA (5% and 10%, w/v, GM-60) and LAP (0.25%, w/v) were gained from Yongqinquan Intelligent Equipment Co., Ltd. (Suzhou, China). The cell-laden bioink with 5 × 10^6^ PDAC cells/mL was transferred into a chute. Five-layer scaffolds (15 mm diameter × 1 mm height, 0.2 mm per layer) were fabricated using a 3D-Bioprint (EFL-BP8601 Ultra, Suzhou, China). The printing parameters were set as follows: speed 10 mm/s, pressure 2 bar and 32°C. The scaffolds were crosslinked by 405 nm ultraviolet at the intensity of 15 mW/cm^2^ for 10 s (5% GelMA, Soft group) or 30 s (10% GelMA, Stiff group), respectively. Then followed by incubation in complete DMEM for 5 days.

### Live/dead staining

Following the manufacturer’s instructions, the live/dead fluorescence staining was executed. Briefly, samples were rinsed with the kit’s assay buffer and then incubated with Calcein-AM for 25 min and Propidium iodide reagent (CA1630, Solarbio, China) for 5 min. Then, further washed and imaged using a confocal microscope (LSM900, Zeiss).

### Phalloidin staining

Fixed cells were permeabilized with 0.3% Triton X-100, then, incubated with phalloidin solution (1:100, CA1610, Solarbio, China). Finally, the above cells were counter stained with DAPI and visualized using a confocal microscopy (LSM900, Zeiss).

### Stiffness measurement

Three-dimensional-bioprinted hydrogel specimens were measured using an electronic universal material testing machine (C42.503, MTS, USA) to determine compressive elastic moduli. The data were calculated according to the slope of the linear portion of the stress–strain curve.

### X-ray irradiation

X-ray irradiation was conducted using a clinical linear accelerator. Following a 5-day culture within the 3D-printed models, cellular samples were exposed to gradient doses of X-rays. The irradiation conditions were: 6 MV energy, 625 MU/min dose rate, at 100 cm source-to-axis distance (SAD), Dose Total (DT) = 0, 2, 4, 6, 8 Gy. The following experiments were conducted 12 h post-irradiation.

### CCK-8 assay

The cultivated cells were harvested and seeded into 96-well plates at a density of 2000 cells per well. After being cultivated for 48 h, 10 μL Cell Counting Kit-8 (CCK-8; AC11L554; Life-iLab, China) solution was added to each well. Finally, the absorbance at 450 nm was detected after incubation for 2 h.

### Colony formation

The density of 1000 cells per well (*n* = 3) were inoculated into 6-well plates. Four percent paraformaldehyde (PFA, ZYFB002-0500, ZUNYAN, NanJing, China) was used to fix colonies following 14 days of incubation. At least 50 cells colored with crystal violet were counted.

### qPCR

TRIzol reagent was utilized to isolate total RNA. cDNA reverse transcription kit (K1622; Thermo Fisher Scientific) was applied to synthesize the template. The gene expression levels were quantified using a real-time PCR system and 2^−ΔΔCt^ method was utilized to determine the relative genes expression levels. The nucleotide primer sequences were displayed in Supplementary Table S1.

### ChIP-qPCR

Chromatin immunoprecipitation-quantitative PCR (ChIP-qPCR) assay kit (Beyotime, P2080S) was applied as per manufacturers’ protocol. Specifically, the nuclear proteins and DNA were crosslinked with 1% formaldehyde, then quenched with glycine and rinsed with ice-cold PBS. After treatment with SDS lysis buffer, the resulting DNA was sheared into short fragments through sonication. H3K18la primary antibody (PTM-1427RM, Jingjie) was incubated and resuspended with Protein A/G magnetic beads overnight with rotation, then, washed the complex. Further, reverse crosslinking, elution and purification of the DNA were performed. IgG was used as a negative control. The ChIP-isolated DNA was subjected to PCR analyses and the primers were listed in Supplementary Table S2. To dissociate the proteins bound to the beads, the washed bead complexes were resuspended in 20 μL loading buffer, then separated by SDS-PAGE. The Fast Silver Stain Kit (P0017S, Beyotime) was utilized to visualize the protein bands.

### Agarose gel assay

Genomic DNA and ChIP-qPCR products fragmented by ultrasonication were size-verified using 1% agarose gel electrophoresis. 1.0 g of agarose powder was dissolved in 100 mL of TAE buffer, then, boiled twice to achieve a homogeneous solution. After cooling slightly, the Gel-Red was added and poured into a mold to solidify. Samples were loaded into the wells, and electrophoresed at 180 V for 15 min. The result was then imaged under a blue light transilluminator for band analysis.

### Immunofluorescence staining

After fixed with 4% PFA, the cells were permeabilized with 0.3% Triton X-100 and blocking with 5% BSA. Primary antibodies: anti-PKM2 (1:50, ab150377, Abcam), anti-LDHA (1:100, ab52488, Abcam), anti-HK2 (1:100, ab209847, Abcam), anti-RAD51 (1:250, ab133534, Abcam) and anti-γ-H2AX (1:250, ab81299, Abcam). Secondary antibodies: goat anti-rabbit IgG H&L Alexa Fluor 647 (1:1000, ab150079, Abcam) and goat anti-rabbit IgG H&L Alexa Fluor 555 (1:1000, ab150078, Abcam). Then, cell nuclei were counter stained with DAPI and imaged by confocal microscopy (LSM900, Zeiss). Fluorescence intensity was quantified with ImageJ.

### RNA-seq and bioinformatics analysis

MIA-PaCa2 cells in the 3D-bioprinted soft and stiff hydrogel models following 5 days culture were conducted with RNA-seq. High-throughput sequencing was performed using the BGISEQ-500 platform (Helix Life, Shanghai, China) with biological triplicates for both groups. DEGs were identified through stringent screening criteria (|Log_2_FC| ≥ 1, *P* < 0.05). The statistically significant DEGs subset underwent comprehensive functional annotation through Gene Ontology (GO) and Kyoto Encyclopedia of Genes and Genomes (KEGG) pathway enrichment analysis. Complementary genome-wide profiling was achieved through the Gene Set Enrichment Analysis (GSEA) Java application. Analytical results were graphically rendered utilizing specialized bioinformatics platforms (https://www.bioinformatics.com.cn).

### Western blot

The supernatant cells lysates were collected and the BCA Protein Assay Kit (ZJ102, Epizyme) were utilized to determine the protein concentration. The total protein was separated by SDS-PAGE and transferred onto PVDF membrane, blocking with nonfat milk and overnight incubation with primary antibodies. anti-PKM2 (1:10 000, ab150377, Abcam), anti-LDHA (1:8000, ab52488, Abcam), anti-HK2 (1:1000, ab209847, Abcam), anti-Pan-Kla (1:1000, PTM-1401RM, Jingjie), anti-H2BK16la (1:1000, PTM-1424RM, Jingjie), anti-H3K9la (1:1000, PTM-1419RM, Jingjie), anti-H3K14 (1:1000, PTM-1414RM, Jingjie), anti-H3K18la (1:1000, PTM-1427RM, Jingjie), anti-H4K5la (1:1000, PTM-1407RM, Jingjie), anti-H4K8la (1:1000, PTM-1415RM, Jingjie), anti-H4K12la (1:1000, PTM-1411RM, Jingjie), anti-H4K16la (1:1000, PTM-1417RM, Jingjie), anti-Histone H3 (1: 1000, PTM-1001RM, Jingjie), anti-Histone H4 (1:1000, PTM-1015RM, Jingjie) and anti-RAD51 (1:30 000, ab133534, Abcam). Next, HRP-Goat Anti-Rabbit Recombinant Secondary Antibody (H+L) (1:5000, ZYID002-0050, ZUNYAN, China) was added to incubate. All antibodies were diluted in antibody dilution buffer (KGC4310-500, KeyGEN BioTECH). The gray bands intensities were quantified using Image J.

### Glucose uptake assay

The supernatant was collected after being centrifuged at 12 000 *g* for 10 min, and glucose concentrations were quantified using a commercial o-toluidine-based glucose assay kit (S0201S, Beyotime, China) as per the manufacturer’s protocol. Absorbance measurements at 630 nm were performed with spectrophotometrically.

### Lactate production

The collected supernatant was measured according to the instruction of the lactic acid assay kit (BC2235, Solarbio, China). The absorbance values were obtained at 570 nm using a spectrophotometer. The amount of lactate was evaluated after normalized according to the cell count.

### Intervention assay

For stiff hydrogel group, PDAC cells were intervened with 2-deoxy-D-glucose (2-DG, 5 mM, HY-13966, MCE), a synthetic analogue of glucose that competitively suppresses glycolysis, blocking cellular energy generation. 20 mM sodium lactate (Nala, HY-B2227B, MCE), an alternative energy and lactate dehydrogenase (LDH) substrate, was also added to the stiff group for 24 h.

### Plasmids transfection

Plasmid transfection was carried out using Lipofectamine™ 3000 (Invitrogen, California, USA) as per supplier’s guidelines. In brief, cells achieved 70–90% confluence and transfected with plasmids. Plasmids and Lipofectamine™ 3000 were incubated with serum-free medium and then added into the respective cell culture dishes. The cells were conventional cultivation for 48 h. ShRNA were provided by Genepharma, and the target sequence for the human shRNA RAD51 were 5′-CTGGATCTATCACAGAAATGT-3′ (1#) and 5′-GCAGTGATGTCCTGGATAATG-3′ (2#). shNC sequence was 5′-GTTCTCCGAACGTGTCACGT-3′.

### Statistical analysis

Data were analyzed using GraphPad Prism 9, with all experiments repeated at least three times independently. Data were presented as the mean ± standard deviation and statistical analysis was performed by Student’s t test. *P < 0.05, **P < 0.01 and ***P < 0.001 were considered to indicate statistically significant differences and ns mean no significant.

## Results and discussion

### Fabrication and characterization of 3D-bioprinted PDAC model with tunable stiffness

The profoundly complex and dynamic PDAC ecosystem consisted of multiple cellular components and displayed various physical traits. PDAC cells resident in a 3D-physiologic niche and sense and respond to omnidirectional mechanical cues. Therefore, constructing a 3D-PDAC model with adjustable mechanics is of significant importance. Emerging bioprinting technology based on controlled deposition enabled the precise encapsulation and essential mechanical support for cells within hydrogel-based matrices, which has been extensively applied to construct various complex structures, such as liver, pancreatic and brain. Recently, solid tumor models based on 3D printing have been applied to explore the cellular behavior and underlying mechanisms. For example, a gastric cancer model with 0.88 mm height and 1.5 cm side length was constructed by gelatin methacrylate hydrogel (GelMA), which accurately replicated the key hallmarks, including invasion, proliferation and the Warburg effect [28]. Breast tumor model with the complex heterogeneity was fabricated based on 3D-bioprinted adipose decellularized extracellular matrix, which utilized to evaluate the drug efficacy through high-throughput screening of various chemotherapeutic drugs [24]. Currently, 3D-printed tumor model with adjustable mechanical properties was utilized to probe the influence of matrix stiffness mediated radioresistance [29], drug response [30] and invasiveness [31]. The neoplasm constructs with tunable mechanical properties have attracted the attention of researchers, which were fabricated via photocurable hydrogels through altering the UV exposure time and concentration. As a typically photo-crosslinkable hydrogel, GelMA was utilized to create tissue-like structures with required shape and size, which harbors the 3D-printed models with fine biocompatible and ideal cell adhesion and proliferation characteristics [32].

**Figure 1:**
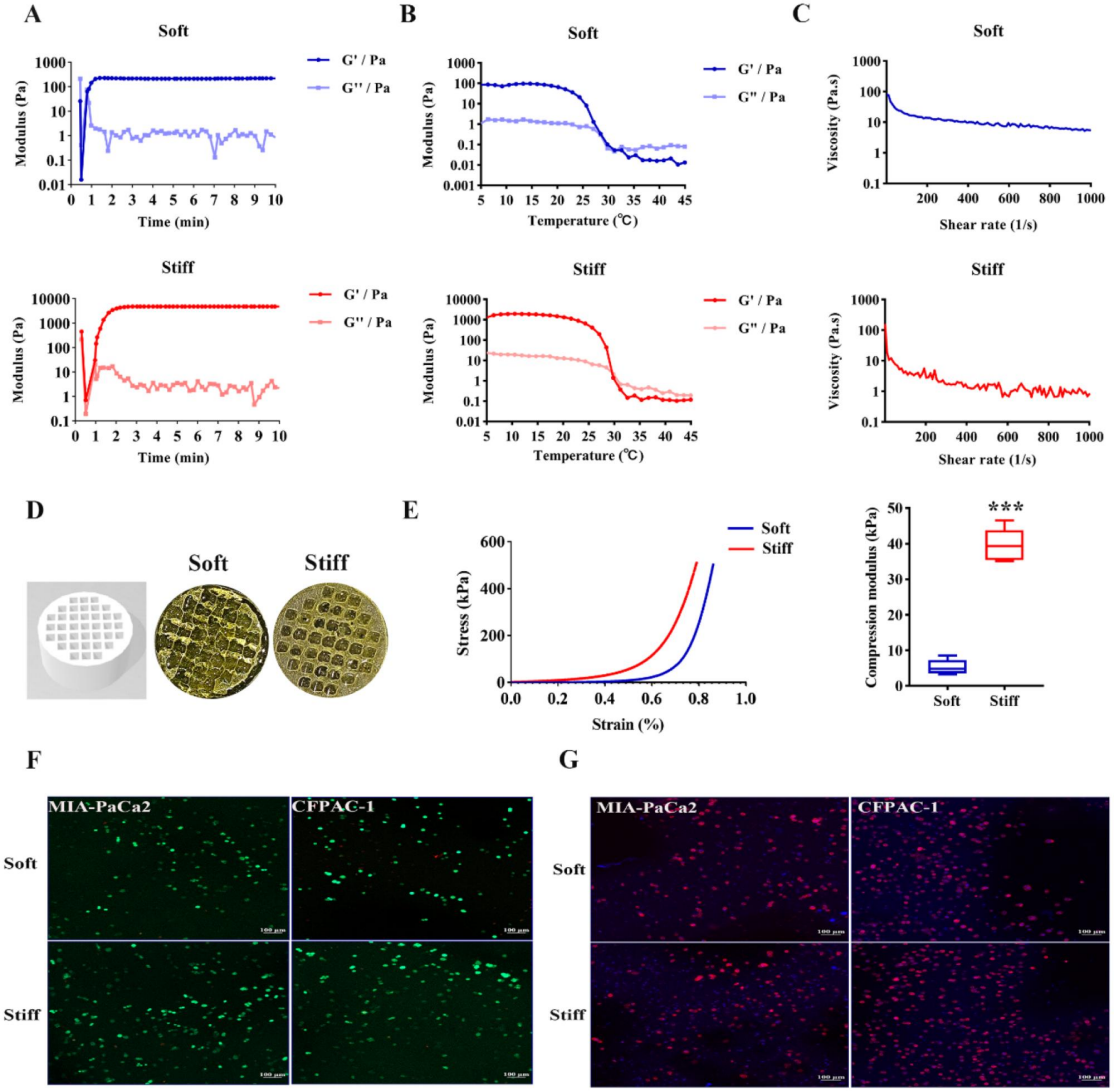
Fabrication and characterization of 3D-bioprinted PDAC models with tunable stiffness. (**A**) The storage modulus (*G*′) and loss modulus (*G*″) as a function of time. (**B**) The *G*′ and *G*″ as a function of temperature. (**C**) Shear-thinning behavior of soft/stiff hydrogel. (**D**) The appearance overview of the soft/stiff hydrogel models. (**E**) Stress (kPa)–strain (%) curve of soft and stiff hydrogels encapsulated with PDAC cells (left). the average apparent Young’s modulus of the two models (right). (**F**) The live/dead staining of 3D-bioprinted tumor model (scale bar: 100 μm). (**G**) Phalloidin-FITC (red) and DAPI (blue) staining of PDAC cells. Scale bar: 100 μm. ***P < 0.001.

Hence, in our research, GelMA hydrogel was harnessed to simulate matrix stiffness of healthy (5%, v/v, Soft group) and PDAC tissues (10%, v/v, Stiff group). Rheological property, the material’s flow and deformation characteristics, was vital during bioprinting as they affect the printing accuracy and structural stability. Consequently, aim to understand the suitability, fidelity and functionality of precise manipulated 3D-printing PDAC model, the rheological property of GelMA bioinks was systematic assessed. Time sweep displayed that the viscous modulus (*G*′) and elastic modulus (*G*″) of 5% and 10% group hydrogels both had stability with the UV exposure time increases (Figure 1A). In the oscillation frequency, the *G*′ and *G*″ of the 10% GelMA hydrogels were higher than the counterpart 5% hydrogel (Supplementary Figure S1A). Meanwhile, both of the two hydrogels showcased thermosensitive according to the temperature sweep, and the intersect of *G*′ and *G*″ indicated the gelation temperature at 32°C (Figure 1B). Steady shear viscosity as a function of shear rate was presented in Figure 1C, and the viscosity declined almost linearly with elevated shear rate, indicative of the excellent shear-thinning property exhibited by both samples. Totally, the above results proved that the hydrogels at the two concentrations with printability. Three-dimensional-bioprinted cell-loading PDAC model was fabricated by mixing the GelMA precursor solution and MIA-PaCa2/CFPAC-1 cells. As shown in Figure 1D, the fabricated hierarchical structures exhibited a uniform porous architecture, with each pore measuring 1.875 mm width and the overall scaffold dimensions of 15 mm × 1 mm (diameter × height), which was conducive to enhancing nutrient diffusion efficiency. In our previous studies, the adjacent and PDAC tissues were gained from Affiliated Hospital of Nantong University and measured the Young’s modulus, which showcased that PDAC tissues with a modulus approximately 40 kPa, significantly higher than normal tissues about 3 kPa [33]. The 5% and 10% GelMA hydrogel models exhibited apparent Young’s moduli of 5.28 ± 2.09 kPa (Soft group), and 39.51 ± 4.66 kPa (Stiff group), respectively (Figure 1E), which were similar to the tissues. As shown in Supplementary Figure S1B, SEM characterization showed differences between the voids of the hydrogels. Then we can observe that the stiff group hydrogels have a denser fiber structure than the soft group hydrogels, and a denser fiber structure means higher stiffness. This also mimics the rigid pancreatic cancer and soft adjacent tissues to some extent. Furthermore, live/dead staining leverages differential cellular permeability and enzymatic activity to distinguish viable from nonviable cells. After cultured for 5 days in the 3D-printed model, the live/dead staining results demonstrated that PDAC cell viability exceeded 90% in both the soft and stiff groups (Figure 1F and Supplementary Figure S1C). Phalloidin staining was performed to visualize F-actin cytoskeletal organization and the result indicated that cells grew spherically and tended to cluster in both of the hydrogels (Figure 1G). In summary, *in vitro* PDAC models with two matrix stiffness gradients was successfully constructed.

### Stiff matrix reinforced radioresistance in 3D-printed PDAC model

Radiotherapy (RT) solely or joint with other antitumor treatment has been clinically utilized to treat over 50% of solid cancers. Due to the ability to induce lethal damage simultaneously prevention adjacent healthy tissues through precise targeting strategies such as intensity-modulated and stereotactic body radiotherapy, RT has been widespread applied. Nevertheless, radioresistance remains main obstacle to improve RT efficacy despite the advanced including more accurate delivery of radiation. Relevant studies showed that radiation-exposed cancer cells-induced DNA breaks via caspase-activated DNase, enhancing radiotherapy sensitivity [34]. Multiple myeloma patient with radioresistance was associated with the upregulation of DNA-PKcs [35]. Furthermore, high expression of glutamine synthetase resulted in apparent diminished radiosensitivity against tumor cell [36]. In solid tumors, high stiffness conferred radioresistance through facilitating DNA double-strand break repair [37]. With the progression of PDAC, the excessive extracellular matrix (ECM) deposition and crosslinking stiffening PDAC setting, whether matrix stiffness influence the radioresistance remain unidentified. In clinical cancer treatment, high radiation doses with the potential to destroy or decelerate tumor growth [38].

**Figure 2:**
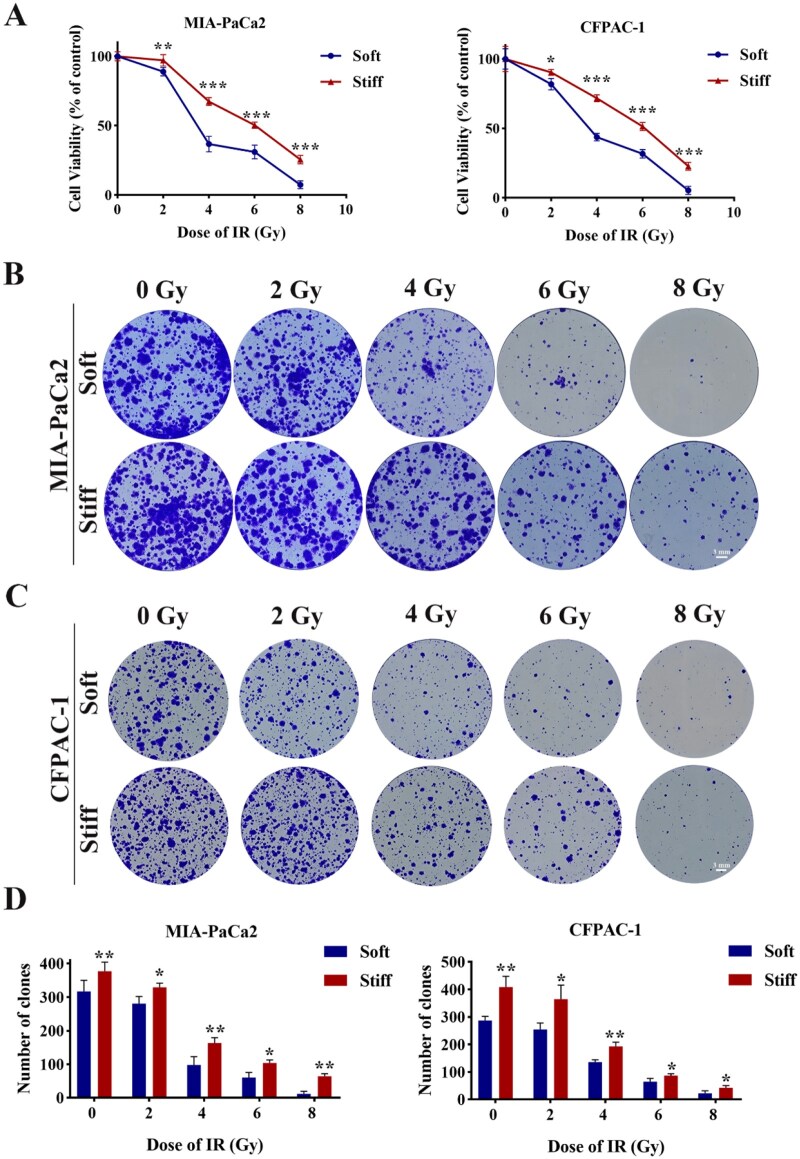
The modulatory role of matrix stiffness on PDAC radiotherapy response with gradient radiation doses. (**A**) The cell viability curve of PDAC cells were exposed to gradient radiation doses in the soft and stiff groups. (**B**, **C**) The colony formation of PDAC cells were exposed to gradient radiation doses in the soft and stiff groups. (**D**) The statistics analysis of clone’s numbers. *P < 0.05, **P < 0.01, ***P < 0.001.

To evaluate the impact of matrix stiffness on radiotherapy response, relevant radiation tests were conducted. The sensitivity to radiation therapy varied significantly among multiple types of tumor cells. Therefore, our study adopted the uniformly increasing gradient irradiation doses at 0, 2, 4, 6, 8 Gy to examine the radiotherapy tolerance of PDAC cells within different stiffness models. The growth curves were generated to systematically assess the impact of varying radiation doses on cancer cell proliferation kinetics both the soft and stiff groups, enabling quantitative evaluation of radiation resistance. The results demonstrated that the stiff group exhibited a higher survival rate compared with the soft group, which illustrated that the stiff group has considerable higher radioresistance (Figure 2A). The clinical observation displayed that desmoplastic PDAC possess dense stroma correlates with poor RT response. The colony formation assay was performed to assess the capacity of single cells to proliferate and form colonies (typically ≥ 50 cells), providing insights into cellular sensitivity to radiation. Additionally, the cloning experiment results demonstrated that radiation sensitivity exhibited a dose-dependent response. Although colony formation decreased sharply with increasing treatment dose in both soft and stiff groups, the stiff group consistently supported a higher number of colonies with larger sizes compared to the soft group. Next, we found that PDAC cells were sensitive to irradiation with lethal dose 50 (DL50) around 4 Gy (Figure 2B–D). These data highlighted matrix stiffness modulated radioresistance. Elevated matrix stiffness upregulates SNAIL1, activating cancer-associated fibroblasts that driven radioresistance through cytokine secretion [39]. Our experimental data further corroborated that high stiffness mediated radioresistance. On the contrary, softer matrices enhanced radioresistance in glioblastoma multiforme, which correlated with chromatin condensation. Matrix stiffness may differentially influence radioresistance depending on disease type.

### Transcriptional profiling demonstrated that glycolysis contributed to matrix stiffness mediated radioresistance

**Figure 3:**
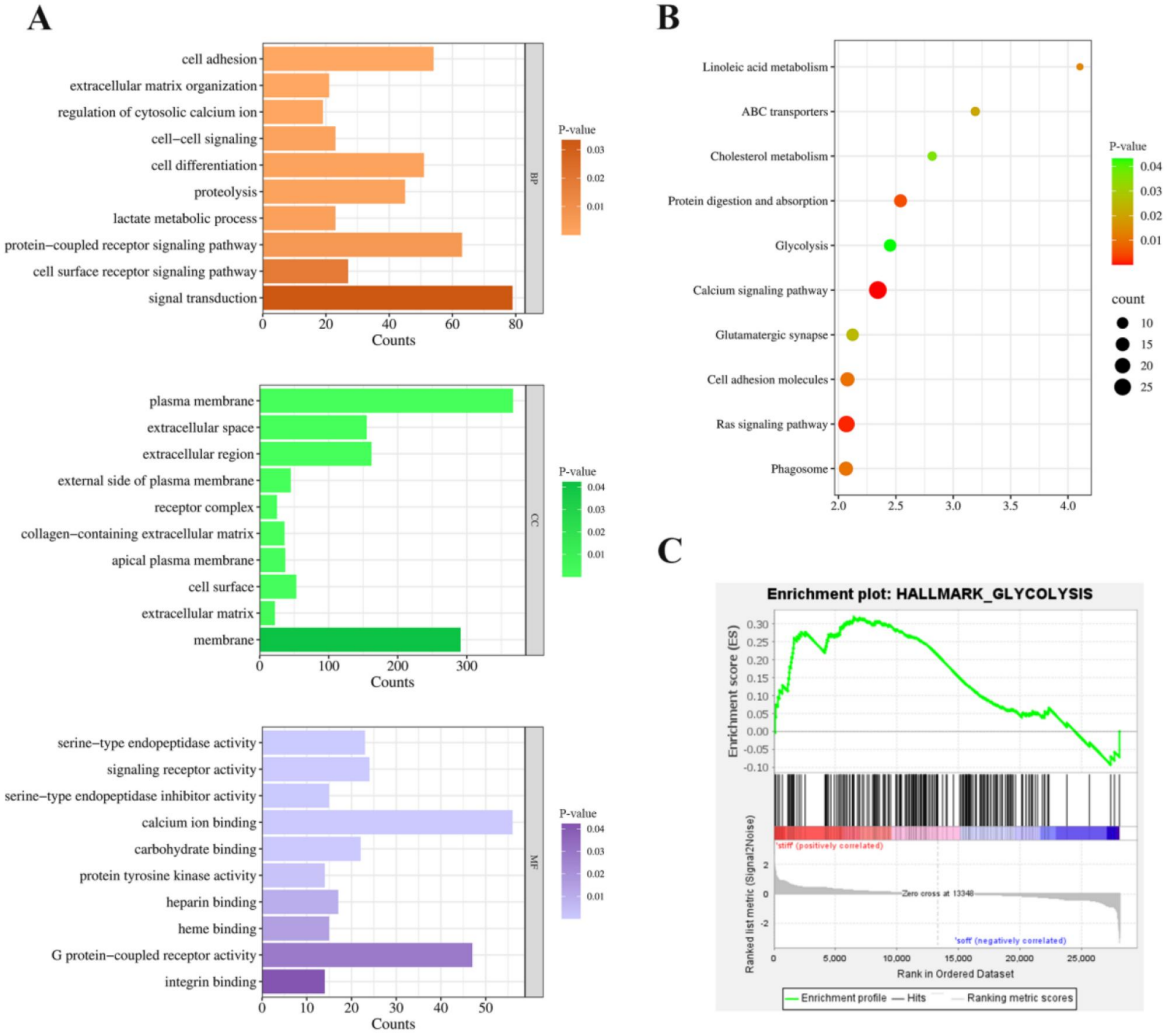
Enrichment analysis based on transcriptional landscape of MIA-PaCa2 cells within 3D-printed matrix stiffness treated with 4 Gy radiation dose. (**A**) The histogram illustrated the top 10 enrichment GO terms related to molecular function (MF, orange), cellular component (CC, green) and biological process (BP, purple) classification of DEGs. (**B**) The bubble chart presented the top 10 KEGG enrichment. (**C**) The glycolysis pathway involved in the GSEA analysis.

To investigate the underlying mechanism of matrix stiffness regulating PDAC radioresistance, cells were exposed to 4 Gy X-ray irradiation from the 3D-printed soft and stiff matrix models were performed with RNA-sequencing. The differentially expressed genes (DEGs) were analyzed through Gene Ontology (GO) and Kyoto Encyclopedia of Genes and Genomes (KEGG) enrichment. GO enrichment analysis revealed distinct stiffness-dependent biological signatures. As seen in Figure 3A, “lactate metabolic process,” “extracellular matrix (ECM) organization” and “signal transduction” were represented in biological processes (BP). The dynamic equilibrium and tight regulation of lactic acid, glycolysis-derived metabolite, are critical for sustaining energy homeostasis, acid-base balance and also served as the therapeutic target in pathological conditions. Simultaneously, lactic acid as a major carbon source for oxidative metabolism through the “lactate shuttle” mechanism, enabling intercellular metabolic coupling [40]. The production of lactate via aerobic glycolysis supported rapid proliferation of cancer cells through maintaining the redox balance and furnishing the biosynthetic intermediates [41]. Additionally, lactate influenced immune cell function and tumor microenvironment remodeling by modulating hypoxia-inducible factor-1 stability and G-protein-coupled receptor mediated pathways [42]. Emerging research highlights lactate’s role as the precursor for histone lactylation, a newly identified post-translational modification that connects cellular metabolism to epigenetic regulation. Lactate-derived lactyl-CoA modifies histone lysine residues (e.g. H3K18la) to activate gene expression involved in wound healing, macrophage polarization and tumor progression [15]. These findings underscore the dual function of lactate as a metabolic intermediate and epigenetic modulator. Cellular component (CC) analysis demonstrated striking enrichment in “collagen-containing ECM” and “focal adhesion,” suggesting their critical roles in ECM remodeling and cell-matrix adhesion-mediated signaling during tumor microenvironment (TME) dysregulation and cancer progression. Molecular function (MF) terms such as “calcium ion binding,” “G-protein-coupled receptor activity” and “integrin binding” were prominent. Ligand binding to the extracellular domain, the largest class of membrane receptors, to activate the downstream effectors, which in turn trigger intracellular signaling pathways involved in regulating diverse cellular processes and external stimuli response (such as matrix stiffness) [43]. Emerging evidence highlighted the role of G-protein signaling in cellular responses to radiation. Overexpression of GRK2 conferred radioresistance in human embryonic kidney cells [44]. G-proteins mediated gamma ray (10 Gy)-induced apoptosis by upregulating Bak expression through activating CREB and AP-1 [45]. The activation of the GPR3β-arrestin2-PKM2 signaling axis in Kupffer cells promotes glycolysis, thereby ameliorating obesity and attenuating hepatic [46]. The interaction of cell-ECM primarily via integrins, the major metazoan adhesion receptors [47]. Preclinical studies showcased that β1 integrin promoted radioresistance in head and neck squamous cell carcinoma, while its inhibition enhanced radiosensitivity [48].

The top 10 KEGG entries were displayed with a bubble chart. The items such as “linoleic acid metabolism,” “cholesterol metabolism” and “glycolysis” were manifested, which revealed that metabolic reprogramming may be involved in matrix stiffness mediated radioresistance (Figure 3B). The enrichment of “ABC transporters” suggested potential drug efflux mechanism contributing to therapy resistance. Staurosporine targeted the ABCA1 protein to reduce the stemness of esophageal squamous cell carcinoma cancer and enhance radiosensitivity [49]. ABCC3 conferred resistance to colorectal cancer radiotherapy by regulating ROS levels and suppressing the caspase-3-dependent apoptotic pathway [50]. Gene set enrichment analysis (GSEA) further confirmed that the striking upregulation of “glycolysis,” “m-TORC1 signaling,” “apoptosis” and “P53 pathway” in the stiff group (Figure 3C and Supplementary Figure S2). The mTORC1 signaling pathway played a central role in con trolling cell growth and metabolism, integrating diverse upstream signals including growth factors, nutrients (amino acids, glucose) and energy status. Follicle-stimulating hormone enhanced glycolytic activity and lactate synthesis by upregulating the key PFKFB3 and LDHA enzymes through mTORC1-dependent signaling [51]. Taken together, the transcriptomic profiling indicated that matrix stiffness may mechanistically drive radiation resistance in PDAC through glycolysis.

### Stiff matrix augmented glycolysis and H3K18la after radiotherapy treatment

**Figure 4:**
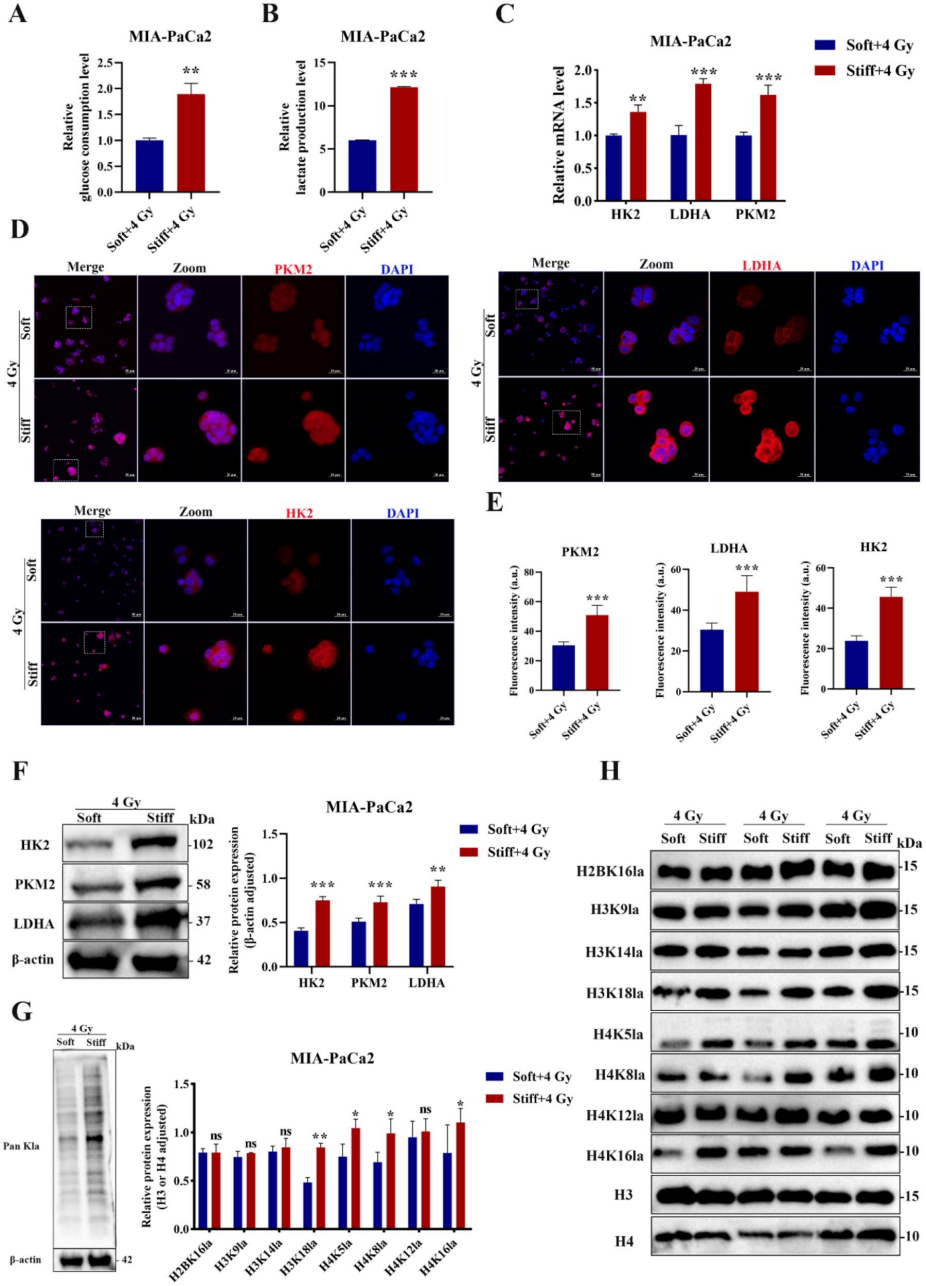
Stiff matrix promoted H3K18la under 4 Gy irradiation dose of MIA-PaCa2 cells. (**A**) The glucose uptake capacity between the soft and stiff groups. (**B**) The lactic acid content determination. (**C**) The relative mRNA expression of PKM2, LDHA and HK2. (**D**) The immunofluorescent images of PKM2, LDHA and HK2 (red: PKM2, LDHA, HK2; blue: DAPI). Scale bar: 50 μm for low magnifications, 20 μm for high magnifications. Zoomed-in areas were shown in white squares. (**E**) Quantitative analysis of PKM2, LDHA and HK2 expression levels. (**F**) The protein expression and statistical analysis of PKM2, LDHA and HK2. (**G**, **H**) The protein expression and statistical analysis of pan-Kla and several site-specific histone lactylation (H2BK16la, H3K9la, H3K14la, H3K18la, H4K5la, H4K8la, H4K12la and H4K16la) in the soft and stiff groups. *P < 0.05, **P < 0.01, ***P < 0.001, ns mean no significance.

As a core hallmark of cancer, metabolic reprogramming sustains a diverse array of cellular processes, including rapid proliferation, invasion and metastasis. Increasing evidences suggest that metabolic rewiring involved in tumor adaptive resistance, while glycolysis recognized as the dominant metabolic reprogramming. The promotion of glycolysis and immunosuppressive cell infiltration drive gemcitabine resistance of pancreatic cancer through PRMT5 stabilization [52]. Glycolytic reprogramming regulated gemcitabine resistance in PDAC through SNRPA/PPA2c/mTORC1 axis [53]. The enhanced glycolytic flux and the upregulation of the pentose phosphate pathway mediated PDAC radioresistance through PDK overexpression [54]. Radiotherapy increased glycolysis and lactate level in pancreatic cancer, which promoted MDSCs functional activation through the GPR81/mTOR/HIF-1α/STAT3 pathway [55]. As a pivotal glycolysis associated enzyme, the knockdown of pyruvate kinase M2 (PKM2) induces radiosensitivity [56]. Thus, the impact of matrix stiffness on glycolysis was investigated in the context of RT. The glucose uptake of PDAC cells cultured in the soft versus stiff matrix was quantified, and validated that the stiff group showed a significantly increased glucose uptake capacity (Figure 4A and Supplementary Figure S3A). During glycolysis process, lactate was generated, a metabolic byproduct and abundant energy source in the tumor environment [57]. Consistently, the intracellular lactate concentration was significantly upregulated in the stiff group versus soft group (Figure 4B and Supplementary Figure S3B). In the presence of ATP, Hexokinase 2 (HK2) catalyzes irreversible phosphorylation of glucose to glucose-6-phosphate, in turn driving glycolysis. PKM2 promotes the conversion of PEP to pyruvate, which facilitates the energy production. Lactate dehydrogenase A (LDHA) leverages NADH as a coenzyme to reduce pyruvate to lactate and regenerate NAD^+^ to maintain continuous glycolysis. Hence, the HK2, PKM2 and LDHA expression were correlated with glycolysis, the results displayed that mRNA level of them in the stiff group (Figure 4C and Supplementary Figure S3C). Meanwhile, both the immunofluorescence (IF) staining and immunoblotting results of HK2, PKM2 and LDHA showcased the similar trend, which all exhibited upregulation in the stiff group (Figure 4D–F). Elevated expression of HK2, PKM2 and LDHA is negatively correlated with overall survival (OS) according to the TCGA and GTEx datasets (Supplementary Figure S4A). In short, matrix stiffness promoted glycolysis under the 4 Gy radiotherapy treatment.

Crucially, elevated intracellular lactate acts as the direct precursor substrate for histone lactylation. The lactyl-CoA derived from lactate is used by writers such as the p300/CBP acetyltransferases to catalyze lactyl modification at the ε‑amino group of lysine residues on histones, including H3K18. This direct mechanistic link, where a metabolite fuels an epigenetic modification, is well-established [15]. Given that lactate as the precursor of histones and non-histones lactylation, and the lactate level differed between the soft and stiff group after 4 Gy RT treatment, thus, the changes in pan-lysine lactylation (pan-Kla) level were measured. Our results demonstrated that the presence of lactylation modifications in both histone and non-histone proteins (Figure 4G). The stiff group exhibited significantly elevated pan-Kla level, with intense signal enrichment observed in the histone region (10–15 kD; Figure 4G). Furthermore, site-specific lactylation on histone H3 and H4 were detected and H3K18la was determined as the most markedly upregulated lactyl-histone site (Figure 4H and Supplementary Figure S4B). H3K18la is a metabolite-dependent epigenetic mark formed by the lactylation of histone H3 at lysine 18 (K18), where lactate is covalently conjugated via an acylation reaction. Related studies have shown that H3K18la potentiated the immune escape of NSCLC cells via activating the POM121/MYC/PD-L1 [58]. H3K18la promoted tumor progression by upregulating VCAM1 to activate AKT-mTOR signaling [59]. Totally, these results demonstrated that H3K18la participated in matrix stiffness-induced radioresistance.

### H3K18la promoted PDAC radioresistance through DNA repair

**Figure 5:**
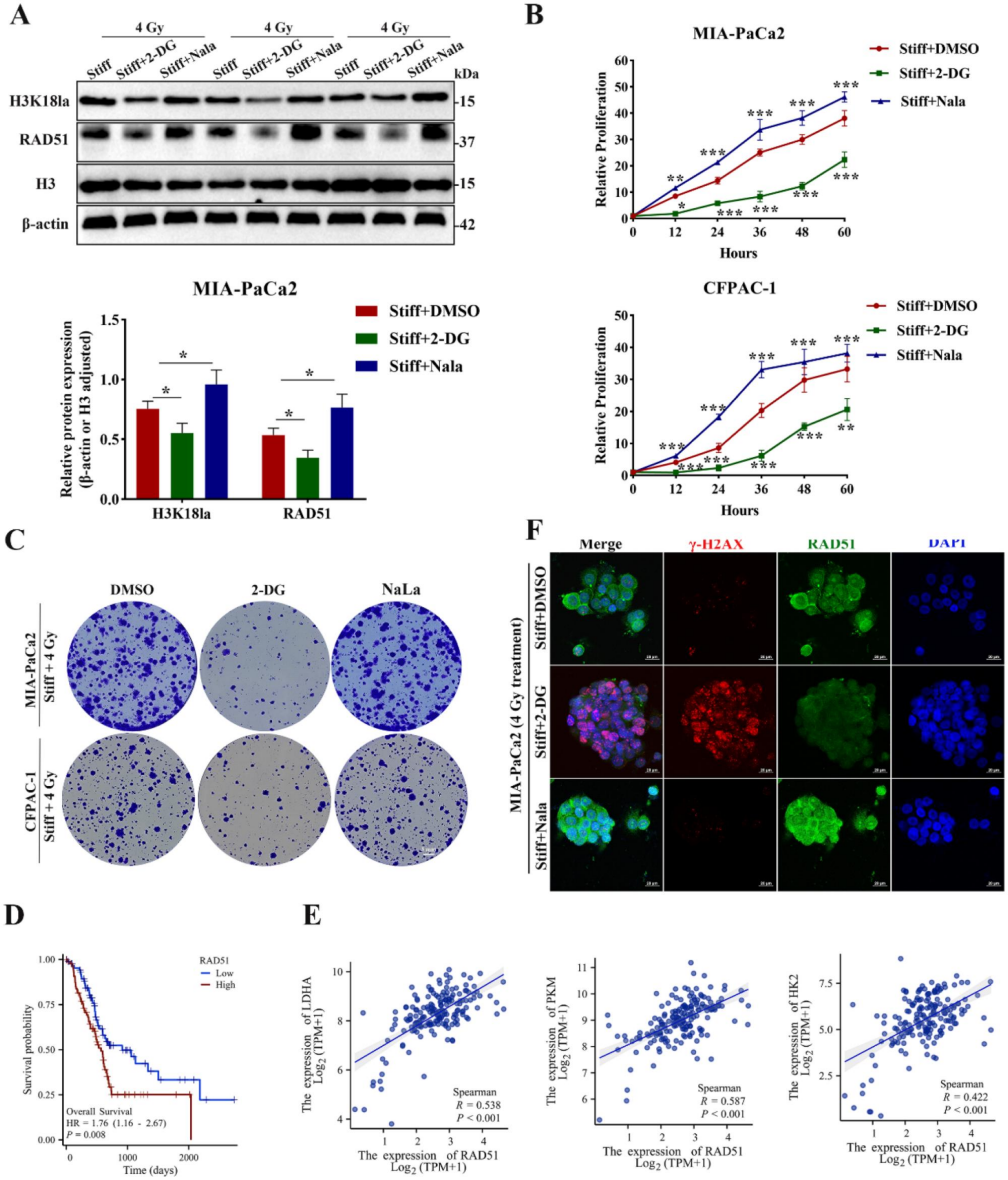
H3K18la augmented radioresistance through transcriptional activation RAD51. PDAC cells were incubated with DMSO, 2-DG or Nala in the stiff group. (**A**) The protein expression and quantitative analysis of H3K18la and RAD51 with three repetitions in MIA-PaCa2 cells. (**B**) The relative proliferation of PDAC cells at designative time points. (**C**) The colony formation images of PDAC cells. (**D**)The overall survival of PDAC patients with low versus high RAD51 expression. (**E**) The correlation between key glycolysis enzymes (HK2, PKM, LDHA) and RAD51. (**F**) Representative immunofluorescent colocalization images of RAD51 and γ-H2AX in MIA-PaCa2 cells (red: γ-H2AX; green: RAD51; blue: DAPI). Scale bar: 20 μm. *P < 0.05, **P < 0.01, ***P < 0.001.

Histone lactylation, a covalent modification characterized by the conjugation of lactate to lysine residues on histones, has been shown to participate in multiple biological processes during tumor progression and therapy resistance. H3K18la accelerated proliferation and radioresistance through enhancing cancer stem cells properties [60]. H3K14la promoted multidrug resistance in hepatocellular carcinoma through the interaction of PTEN with ubiquitin E3 ligase NEDD4 [61]. H3K18la promoted carcinoma migration, invasion and EMT in head and neck squamous cell [62]. As a direct modification marker of lactate, H3K18la directly “writes” metabolic reprogramming signals into chromatin, thereby regulating gene expression. To verify whether H3K18la was involved in matrix stiffness governed radioresistance, the stiff group was supplemented with 2-Deoxy-D-glucose (2-DG, glycolysis inhibitor) or added exogenous lactate (sodium lactate, Nala). The results portrayed that 2-DG treatment reduced the intracellular lactate accumulation and suppressed H3K18la, while for the addition of Nala promoted the H3K18la level (Figure 5A). Furthermore, survival rates of PDAC cells in the stiff group treated with 2-DG or Nala were compared, DMSO group as control. The results showcased that Stiff + 2-DG group diminished the proliferation of PDAC cells, while Stiff + Nala group promoted the proliferation (Figure 5B). The colony formation assay revealed that colony numbers of Stiff + 2-DG group exhibited the lowest, whereas the Stiff + Nala group showcased a strikingly increase both compared with the DMSO group (Figure 5C and Supplementary Figure S4C). Collectively, H3K18la level played an essential role in matrix stiffness regulated radioresistance. RT-induced DNA damage commonly triggered the activation of several cell-programmed responses to maintain genomic stability [63]. E2F transcription factor dynamically orchestrated DNA damage repair by modulating the key repair genes and checkpoint regulators. Vorinostat suppressed BRCA1 and RAD51 expression by disrupting E2F-mediated transcriptional regulation, highlighting the critical role of E2F in repair capacity [64]. The G2/M checkpoint primarily served to establish a transient temporal window for DNA damage surveillance and repair via halting the G2-to-M phase transition of the cell cycle, which ensuring genomic integrity. The terms of “DNA repair,” “E2F targets” and “G2M checkpoint” were also enrichment with GSEA analysis in the stiff group (Supplementary Figure S5). Thus, we speculated that matrix stiffness-induced histone lactylation drive radiotherapy resistance by DNA repair.

### H3K18la transcriptionally upregulated RAD51 expression to facilitate DNA repair and enhance PDAC radioresistance

Representing a novel post-translational modification, histone lactylation has functioned as a dynamic regulator of gene expression. The lactate modification of histone, particularly at H3K18 site (H3K18la), has been validated to link cellular metabolic state to epigenetic reprogramming. H3K18la deposition at anti-inflammatory/repair gene promoter of M1 macrophages, which balanced the immune response and tissue homeostasis [15]. In hepatocellular carcinoma, H3K18la enhanced the NFS1 transcription, which decreased cellular sensitivity to ferroptosis [65]. The H3K9la enrichment at the promoter region of homologous recombination (HR) repair genes (e.g. RAD51 and BRCA2) promoted their transcriptional activation, thereby enhanced DNA damage repair capacity and diminished the cytotoxic efficacy of cisplatin [25]. RAD51 regulated the DNA repair via HR, which prompted the restart of replication and provided error-free repair of double-strand break (DSB). The western blot results showcased that lactylation suppression with 2-DG significantly reduced RAD51 level, while Nala treatment upregulated the RAD51 expression (Figure 5A). Through the TCGA and GTEx datasets, the log-rank test analysis demonstrated that OS was significantly shorter in the RAD51-high group than in the RAD51-low group (Figure 5D). Further, the expression correlation was analyzed, and the results displayed that HK2, PKM, LDHA expression were positively correlated with RAD51 (Figure 5E). As radiotherapy primarily induced DSBs to eradicate cancer cells, the extent of DNA damage directly reflected the degree of radioresistance. As the phosphorylated form of the histone variant H2AX at serine 139 (S139), γ-H2AX formed discrete nuclear foci at DSB sites, acting as marker for DNA damage and recruit repair proteins. IF colocalization analysis of RAD51 and γ-H2AX demonstrated that Stiff + 2-DG group significantly reduced RAD51 expression and elevated γ-H2AX foci formation, whereas Stiff + Nala group exhibited minimal alteration both compared to the DMSO group (Figure 5F and Supplementary Figures S4D and S6).

**Figure 6:**
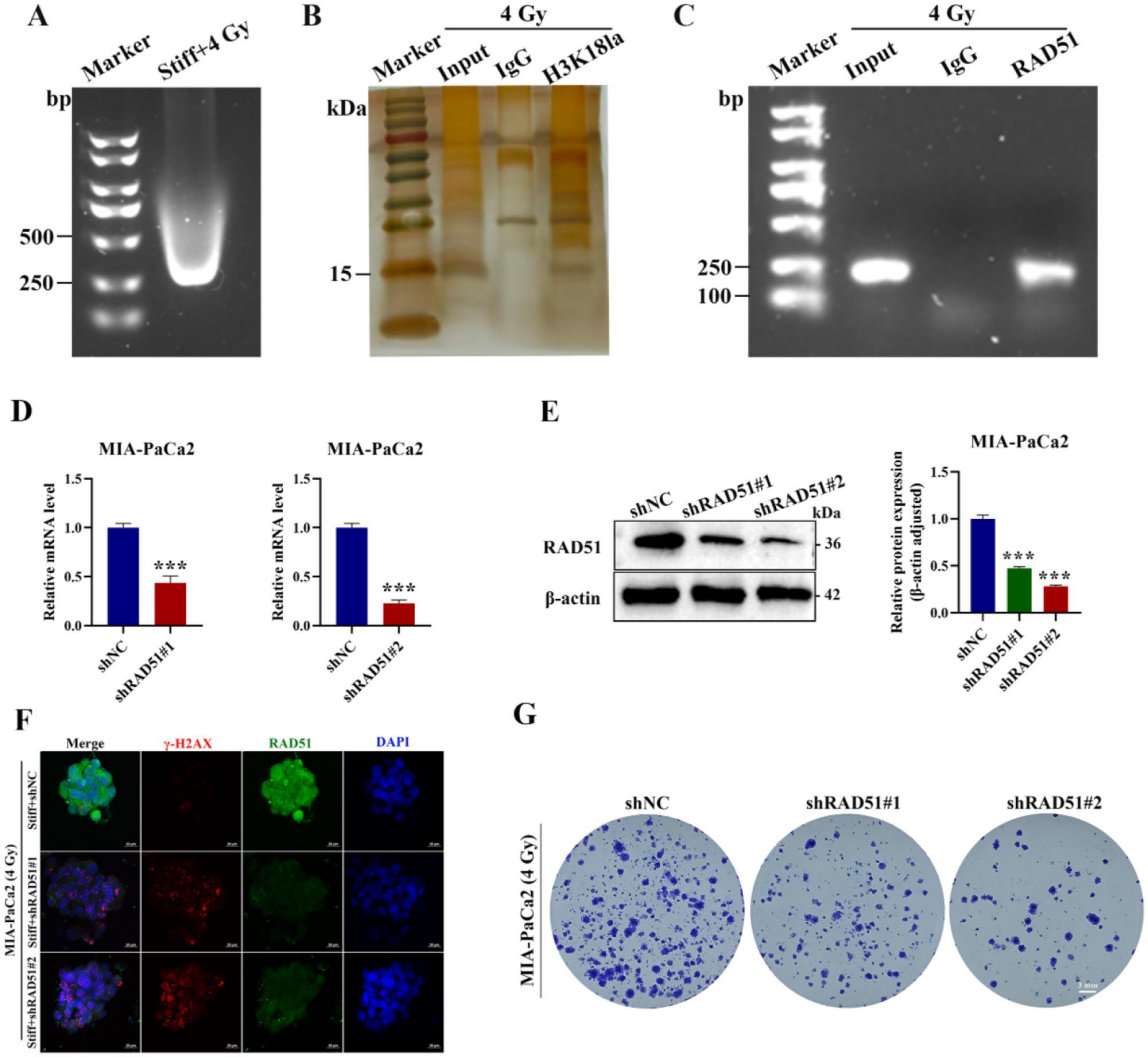
RAD51 involved in PDAC radioresistance via regulating DNA damage repair. (**A**) Ultrasonic effect testing. (**B**) Silver staining of proteins separated by SDS-PAGE. (**C**) Evaluation of DNA amplification efficiency via agarose gel electrophoresis after ChIP-PCR. (**D**, **E**) Probed the RAD51 knockdown efficiency from the gene and protein level. (**F**) Representative immunofluorescent colocalization images of RAD51 and γ-H2AX (red: γ-H2AX; green: RAD51; blue: DAPI). Scale bar: 20 μm. (**G**) Colony formation with RAD51 knockdown. ***P < 0.001.

To investigate the function of histone lactylation, we conducted chromatin immunoprecipitation-quantitative PCR (ChIP-qPCR) with an anti-H3K18la antibody to interrogate whether this specific histone modification was enriched at the RAD51 promoter. Chromatin was subjected to sonication to produce DNA fragments of 250–500 bp in length (Figure 6A), followed by ChIP. Silver staining of the immunoprecipitated chromatin fractions from the ChIP assay, compared to the Input control, revealed a specific H3K18la band, confirming the successful enrichment of this histone modification (Figure 6B). The enriched DNA fragments from the immunoprecipitation were amplified by PCR and analyzed by agarose gel electrophoresis. The results demonstrated a significantly higher enrichment of DNA regions associated with the RAD51 gene in the H3K18la group immunoprecipitated with the target-specific antibody compared to the control group using nonspecific IgG antibody (Figure 6C). Taken together, histone lactylation regulated RAD51 transcriptional activation. Recent researches have uncovered synergistic and competitive interactions among histone modifications. For instance, H3K18la and H3K9la co-enrich with the canonical active mark H3K27ac at promoter regions, synergistically promoting the transcriptional initiation of effector genes during CD8^+^ T cell activation [66]. In hepatic stellate cell (HSC) activation, histone lactylation competes with acetylation, which explains why class I histone deacetylase (HDAC) inhibitors impede HSC activation [67]. Collectively, histone lactylation participates in a sophisticated and dynamic epigenetic circuitry, via mechanisms ranging from competing for shared lysine residues to cooperating with other active marks at regulatory elements. We explicitly stated that our data established a clear link between H3K18la and RAD51 expression, the potential interplay with other relevant histone modifications, including but not limited to H3K27ac and H3K9la was not addressed in this study and represents an important and interesting direction for future research. To further validated whether the RAD51 regulates radioresistance by facilitating DNA damage repair, shRAD51 experiment was performed to evaluate γ-H2AX foci and clonogenic survival. The RAD51 expression was reduced after knockdown assay in the stiff group (Figure 6D and E). Notably, IF co-staining of RAD51 and γ-H2AX showed that lower RAD51 region with high γ-H2AX foci (Figure 6F and Supplementary Figure S7A). RAD51 knockdown led to an increase in γ-H2AX foci and the reduction in colony formation (Figure 6G and Supplementary Figure S7B). Collectively, these findings presented that histone lactylation (H3K18la)-driven RAD51 activation repaired radiotherapy-induced DNA damage.

## Conclusion

Taken together, our research aims to molecular mechanism underlying matrix stiffness mediated PDAC radioresistance. First, the adjustable matrix stiffness resembled the rigidity of healthy and pathology tissues were successfully constructed based on 3D-printed GelMA hydrogel, with stiff matrix boosted radioresistance. Further, we validated that glycolysis and histone lactylation (H3K18la) participated in the radioresistance event. Additionally, H3K18la mediated RAD51 transcriptional regulation to repair DNA damage. Collectively, the depth mechanism that matrix stiffness triggered H3K18la to enhance RAD51 transcriptional activation and DNA reparation was uncovered, which played an essential role in PDAC radioresistance. We propose that targeting the “stiffness-glycolysis-H3K18la-RAD51” axis could be a novel strategy to overcome radioresistance. However, directly targeting a specific histone modification remains a significant challenge. The primary limitation lies in the current lack of highly specific small-molecule inhibitors that can selectively target the “writer” (e.g. acyltransferases for H3K18la), “eraser” or “reader” proteins of H3K18la without affecting other closely related epigenetic pathways (such as acetylation). However, we proposed that combining glycolysis inhibitors (e.g. LDHA inhibitors) or drugs that disrupt lactate production/transport with radiotherapy could be a potent strategy to suppress H3K18la levels and radiosensitize PDAC. From a biomechanical perspective, this study offers novel perspectives for addressing radioresistance in PDAC. Reducing tumor stiffness through the targeted delivery of agents like stromal enzymes or mechano-targeting inhibitors represents a viable strategy for enhancing radiosensitivity. The combination of these modalities is anticipated to achieve dual efficacy by simultaneously overcoming the biomechanical barriers and intrinsic radioresistance of PDAC, ultimately leading to superior treatment outcomes.

## Supplementary Material

rbag071_Supplementary_Data

## Data Availability

Data will be made available on request.
