# Supplementary material for: Three-dimensional-bioprinted stiff matrix triggers PDAC radioresistance through histone H3 lysine 18 lactylation (H3K18la) potentiates RAD51 activation
Source: Regen Biomater. 2026 Apr 10;13:rbag071. doi: 10.1093/rb/rbag071 (PMC13157222; doi:10.1093/rb/rbag071)
Supplement: rbag071_Supplementary_Data [file rbag071_supplementary_data.zip › Supporting information for publication.docx]

**3D-bioprinted stiff matrix triggers PDAC radioresistance through histone H3 lysine 18 lactylation (H3K18la) potentiates RAD51 activation**

Xue Zhang^a, b #^, Hongyu Zhu^a, c #^, Zihan Shi^a, d #^, Yifei Lu^a^, Mingyue Chang^b^, Yan Li^a^, Yahong Zhao^b^_,_ Yumin Yang^b **^, Yibing Guo^a *^

^a^ Research Center of Clinical Medicine, Affiliated Hospital of Nantong University, Nantong University, 226001 Nantong, Jiangsu, China.

^b^ Jiangsu Key Laboratory of Tissue Engineering and Neuroregeneration, Co-innovation Center of Neuroregeneration, Nantong University, Nantong, P. R. China.

^c^ Department of Trauma Center, Affiliated Hospital of Nantong University, 226001 Nantong, Jiangsu, China.

^d^ Department of Gastroenterology, Affiliated Hospital of Nantong University, 226001 Nantong, Jiangsu, China.

Correspondence address: Tel: +86-513-85052612, E-mail: guoyibing2008@163.com (Y.G.); Tel: +86-513-85511585, E-mail: yangym@ntu.edu.cn (Y.Y.)

^#^ These authors contributed equally to this work.

**
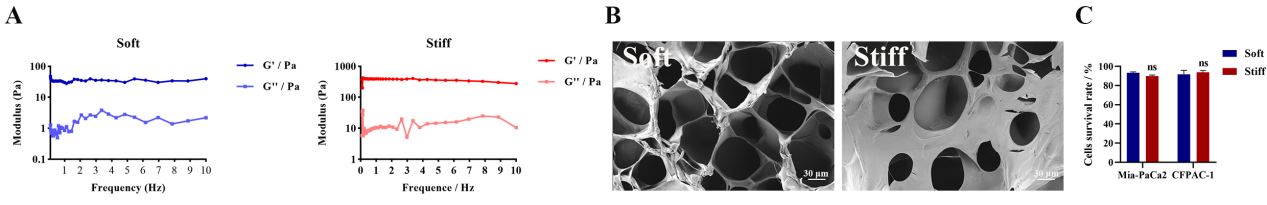
**

**Fig. S1 The measurement of storage modulus (G') and loss modulus (G****") against the frequency, SEM images of GelMA with tunable stiffness and cell viability of PDAC.** Scale bar: 30 μm. **p* < 0.05, ***p* < 0.01, ****p* < 0.001, ns mean no significance.

**
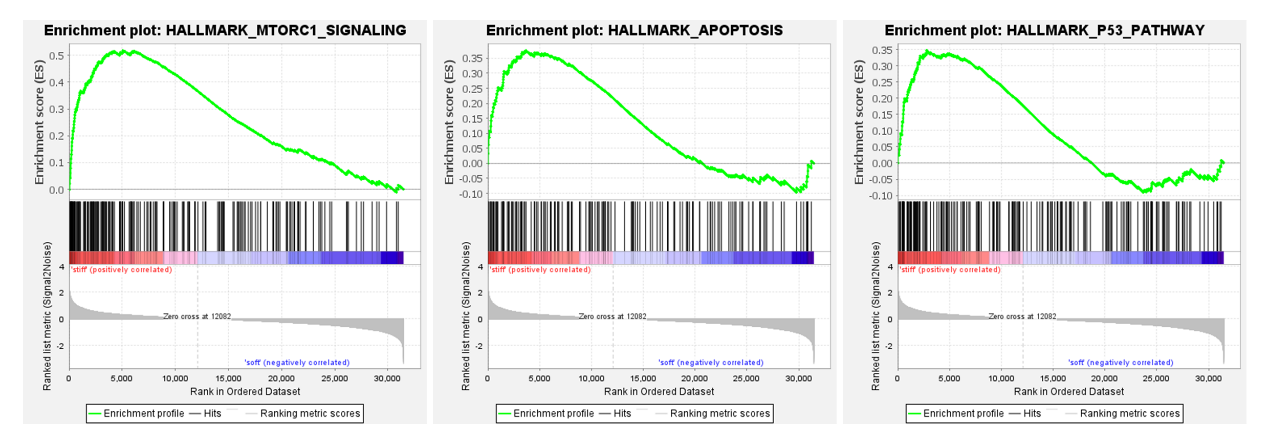
**

**Fig. S2 The GSEA term of "m-TORC1 signaling", "apoptosis" and "P53 pathway" in the stiff group.**

**
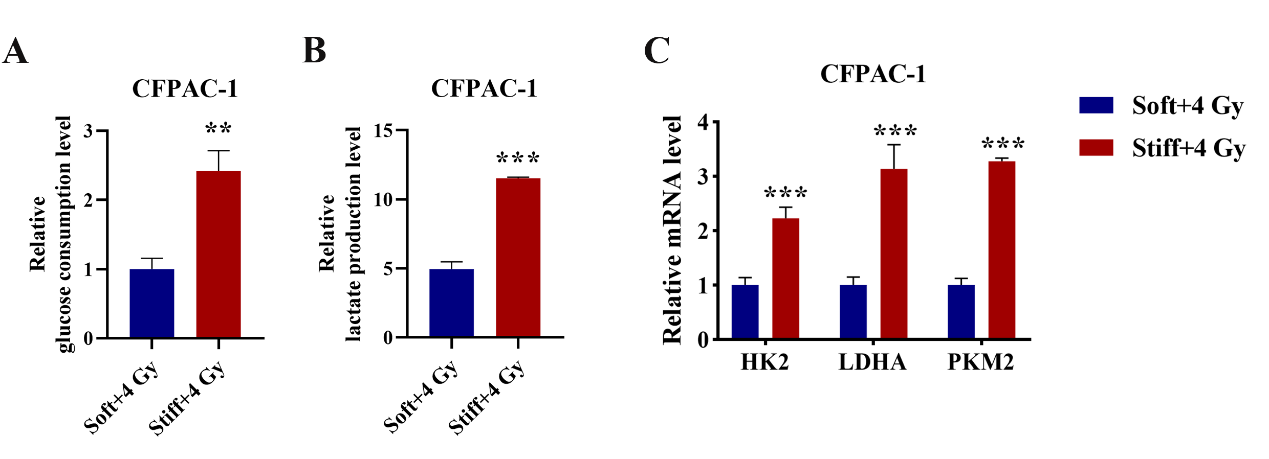
**

**Fig. S3 Stiff matrix promoted glycolysis u****nder 4 Gy irradiation dose of CFPAC-1 cells.** (A) The glucose uptake capacity between the soft and stiff groups. (B) The lactic acid content determination. (C) The relative mRNA expression of PKM2, LDHA and HK2. **p* < 0.05, ***p* < 0.01, ****p* < 0.001.


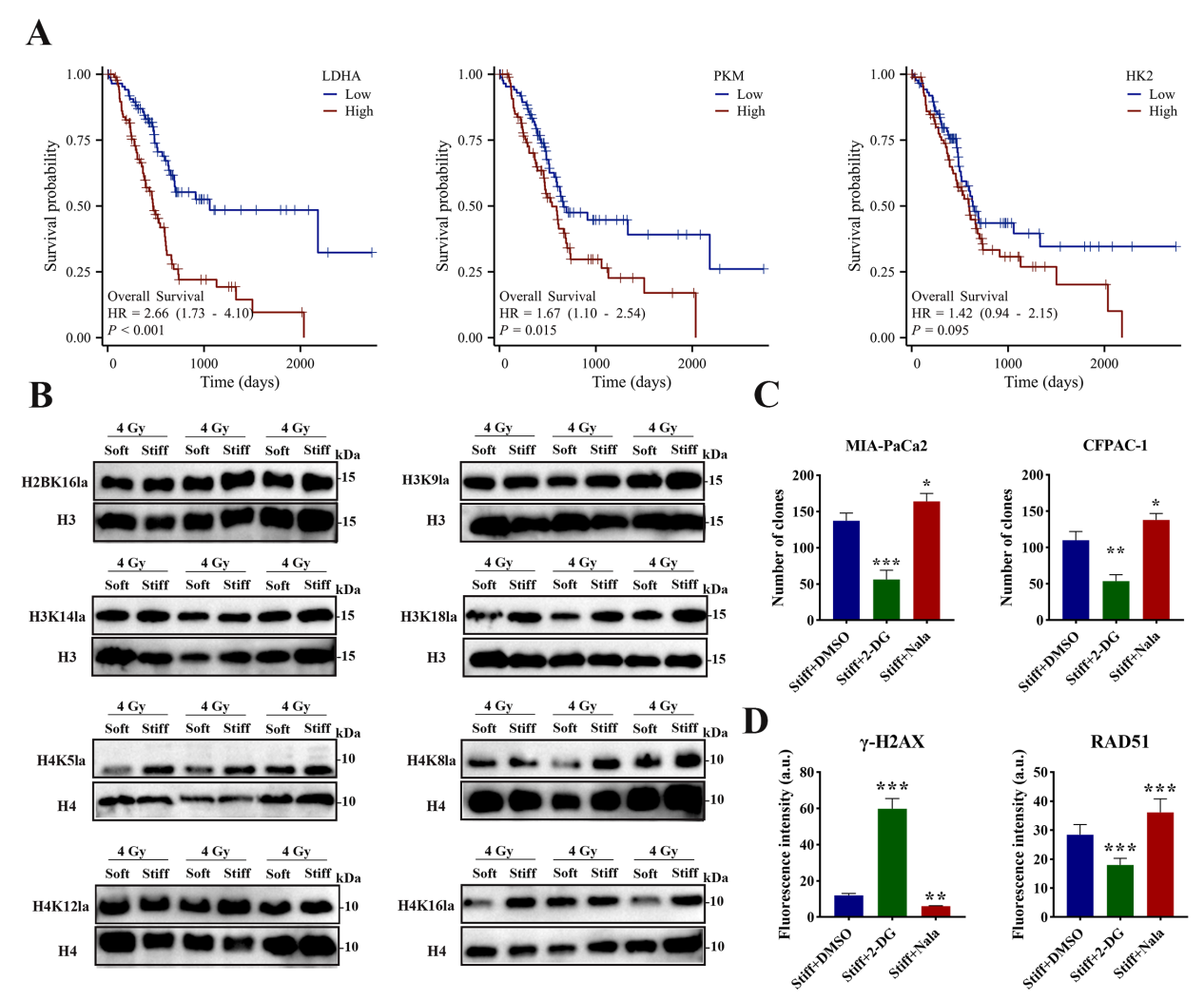


**Fig. S4 The overall survival and statistics analysis.** (A)The overall survival of PDAC patients with glycolysis related genes (LDHA, PKM and HK2) low and high expression. (B) of several site-specific histone lactylation (H2BK16la, H3K9la, H3K14la, H3K18la, H4K5la, H4K8la, H4K12la and H4K16la) in the soft and stiff groups. (C) The statistics analysis of clones numbers. (D) Quantitative analysis of γ-H2AX and RAD51 expression levels. **p* < 0.05, ***p* < 0.01, ****p* < 0.001.


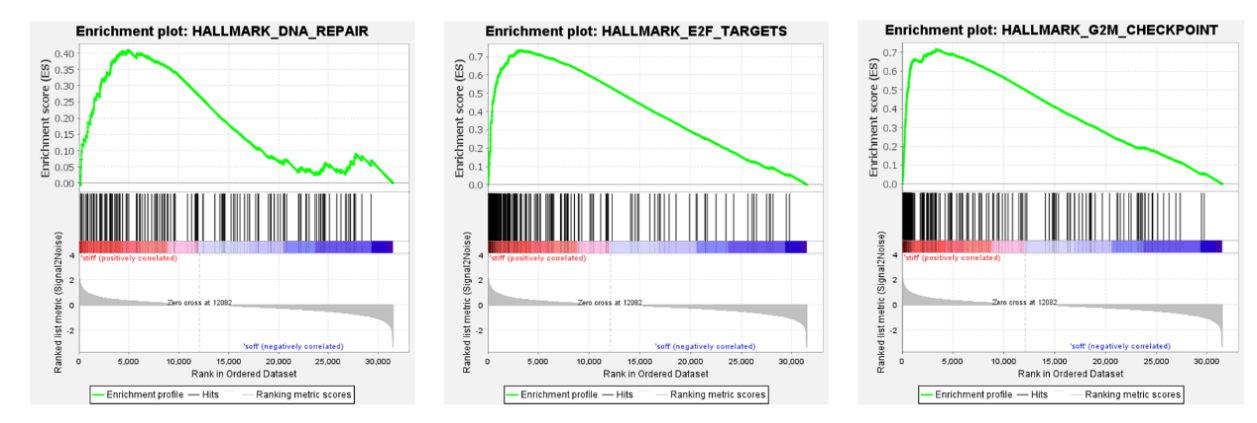


**Fig. S5 GSEA revealed significant enrichment of “DNA repair”, “E2F targets” and “G2M checkpoint” in the stiff group.**


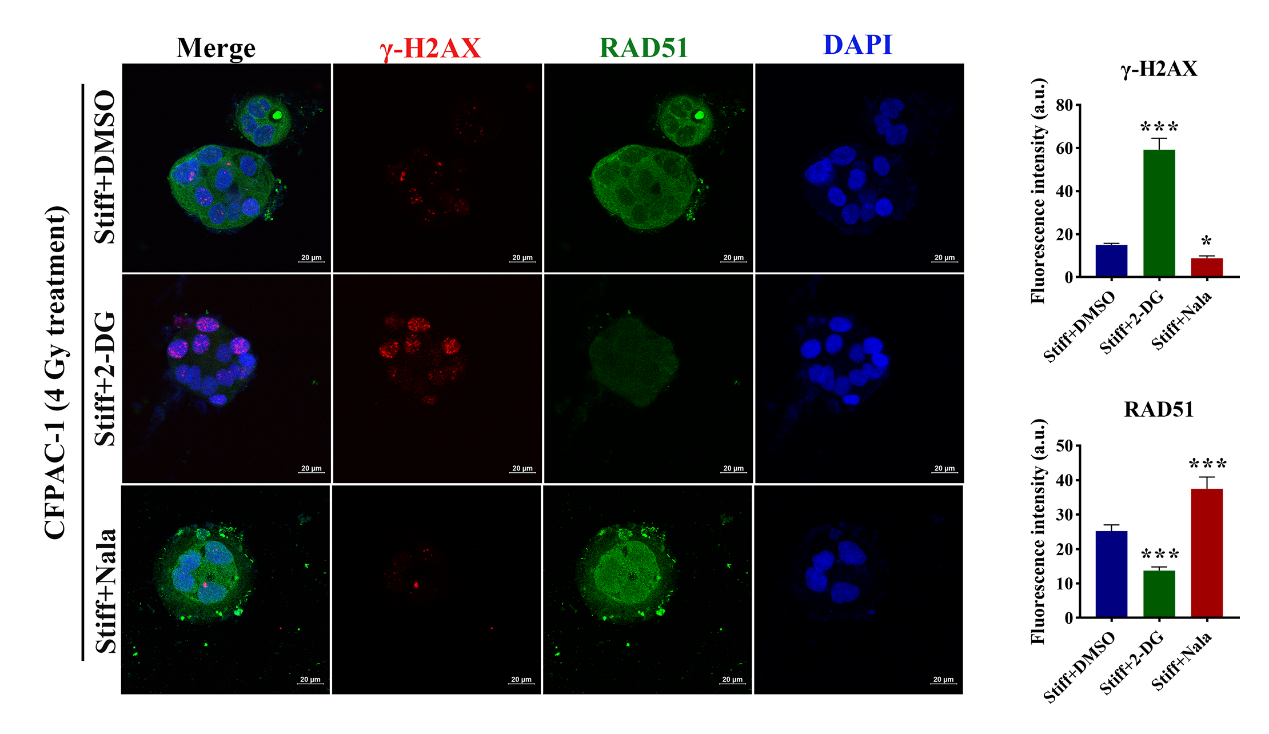


**Fig. S6 Representative immunofluorescent colocalization images and quantitative analysis of RAD51 and γ-H2AX in CFPAC-1 cells** **(red: γ-H2AX; green: RAD51; blue: DAPI). Scale bar: 20 μm.** **p* < 0.05, ***p* < 0.01, ****p* < 0.001.

**
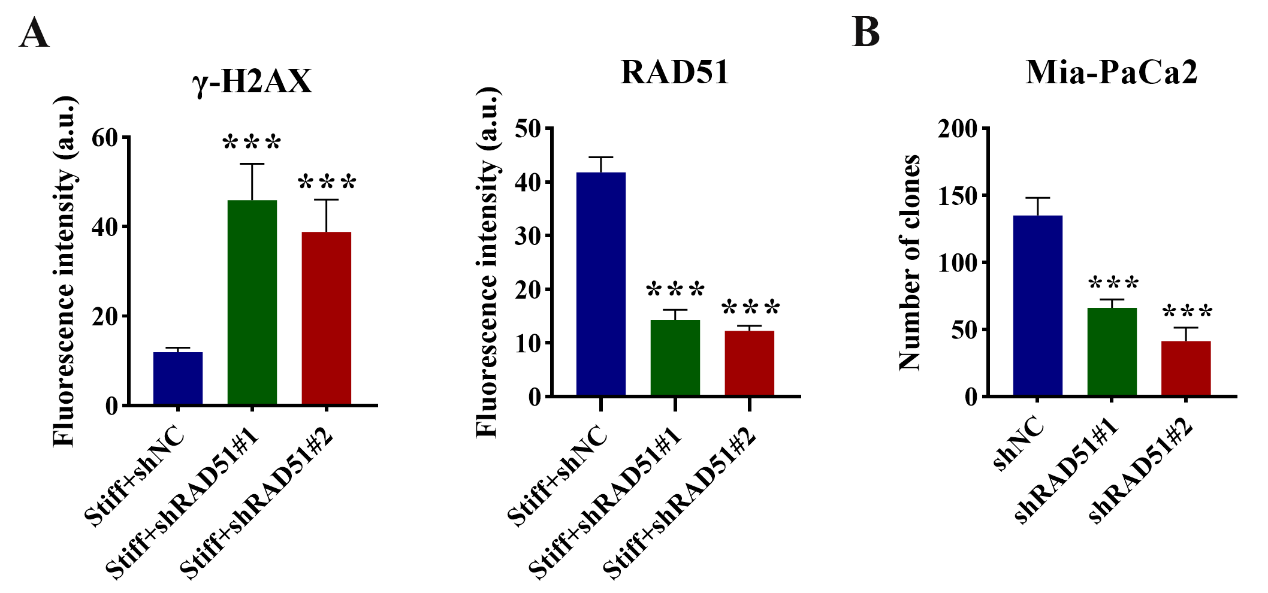
**

**Fig. S7 The quantitative analysis of γ-H2AX and RAD51 expression levels and statistics analysis of clones number.** **p* < 0.05, ***p* < 0.01, ****p* < 0.001.

**Table. S1**

| **Gene** | **Sequence (5’ - 3’)** | **Length** |
| --- | --- | --- |
| **β-Actin** | **F CGGAGGAACCACCATGTACC**  **R GCAGCCTTCACAGAGGCAAAT** | **20**  **21** |
| **HK2** | **F GATTGCCTCGCATCTGCTTG**  **R GCTCCAAGCCCTTTCTCCA** | **20**  **19** |
| **LDHA** | **F CAGCCCGATTCCGTTACCTAATGG**  **R ACACCAGCAACATTCATTCCACTCC** | **24**  **25** |
| **PKM2** | **F AGAAAGGTGCCGACTTCCTG**  **R GCTCGACCCCAAACTTCAGA** | **20**  **20** |
| **RAD51** | **F TAAGTCCAGCTTGGCCAAGG**  **R AGTAGCTGGGACTACAGGCA** | **20**  **20** |

**Table. S1 Primer sequence of β-Actin, HK2, LDHA, PKM2 and RAD51.**

**Table. S2**

| **Target Gene** | **Sequence (5’ - 3’)** | **Length** |
| --- | --- | --- |
| **RAD51** | **F ATTACAGGCATGCACCACCA**  **R CATGTAAGGAGAGGCCAGGC** | **20**  **20** |

**Table. S2 Primers sequence in ChIP-qPCR validation.**
